# Choline: An Essential Nutrient for Human Health

**DOI:** 10.3390/nu15132900

**Published:** 2023-06-27

**Authors:** Milagros Gallo, Fernando Gámiz

**Affiliations:** 1 Departamento de Psicobiología, Instituto de Neurociencias, Centro de Investigación Biomédica, Universidad de Granada, 18071 Granada, Spain; fernandogamiz@ugr.es; 2 Instituto de Investigación Biosanitaria ibs, 18012 Granada, Spain

Choline is an essential nutrient that plays a role in the synthesis of the phospholipid membrane, critical for cell functions, and it is the major source of methyl donors relevant for epigenetic modifications of the genome. It is also the precursor of the neurotransmitter acetylcholine. Thus, choline is involved in several functions relevant for maintaining human health from early life to aging. The dysregulation of choline-related functions has therefore been described in several clinical conditions. Since dietary choline availability modulates choline levels by complementing endogenous synthesis, understanding its metabolic pathways and action mechanisms might contribute to the design of nutritional interventions as promising treatments.

This Special Issue covers research on the involvement of choline in a wide range of biological systems whose dysregulation is associated with various disorders, including metabolic syndrome [1], obesity [2], microbial dysbiosis [3], celiac disease [4], neurological disorders [5], and inflammatory muscle diseases [6], as well as chronic cardiovascular and kidney diseases, among others. Two systematic reviews deal in depth with the contribution of choline to the structure and function of skeletal muscles [6] and the brain [5]. An additional review covers the relationship between gut microbiota and choline metabolism [3]. Three experimental papers report the potential relevance of advancing knowledge on choline metabolism for developing novel biomarkers and treatments of several diseases, such as celiac disease [4], metabolic syndrome [1] and obesity [2].

Choline contributes to the skeletal muscle structure and function due to its role in phospholipid synthesis, which is critical for the cell membrane composition, and also as the precursor of the neurotransmitter acetylcholine, which triggers contraction [6]. In vitro, preclinical and clinical studies confirm the effects of choline supplementation and deficiency on muscle fat and protein metabolism, as well as regulation of intercellular homeostasis. Adequate dietary choline intake regulates fat and protein metabolism, decreasing fatty acids synthesis, and counteracts inflammatory responses, apoptosis, and autophagy. Attention has been drawn to the need for more research on the clinical consequences of choline supplementation in human skeletal muscle structure and function, given that evidence supporting its relevance comes mainly from basic research [6].

The effect of dietary choline availability on brain development and function is widely accepted since animal and human research has provided evidence supporting the neuroprotective and cognitive-enhancing effects of choline dietary supplementation at different developmental stages. A systematic review of rodent studies performed during the last two decades indicates that cognitive improvement induced by choline supplementation has mainly been attributed to enhanced cholinergic neurotransmission in the hippocampal system [5]. Accordingly, most of the studies have evaluated the effects of choline supplementation and deficiency in hippocampal-dependent learning and memory tasks and the related impact on hippocampal neurogenesis, brain choline levels, cholinergic receptors, and intracellular pathways activated by acetylcholine. This seems to be related to the focus on the hippocampal system as critical for conscious/declarative memory. Gámiz and Gallo [5], however, point to the relevance of exploring other learning systems as well as using a variety of attention and emotional tasks, given the involvement of the cholinergic neurotransmission in other brain systems. Furthermore, other choline functions beyond its being a precursor of acetylcholine remain widely unexplored.

Advancing knowledge of the relationship between dietary choline and microbiota composition is most relevant for understanding its impact on health. Choline is metabolized to trimethylamine (TMA) by gut microbiota in the large intestine and delivered to the liver through the portal circulation system. Individual differences in microbiota composition influence TMA production, which is associated with higher activity of particular phyla such as Firmicutes and Proteobacteria. Hence, using these bacteria as probiotics is a potential tool to regulate dietary choline effects on health. Moreover, changes in dietary choline levels modify the gut microbiota composition, and choline-deficient diets might lead to microbiota alterations and health problems. A review of the present knowledge on the specific microbial enzymes involved in TMA production pathways and the microbes carrying the responsible genes has shown that it is necessary to pay attention to host genetics, co-metabolism and diet in addition to gut microbiota [3].

Likewise, the proposed link between inadequate levels of dietary choline and the risk of pathological conditions such as cardiovascular disease and type-2 diabetes that are associated with obesity and the metabolic syndrome is of great importance to favor inexpensive and affordable preventive health care.

DiBella and colleagues [1] performed a study to explore the effect of choline supplementation and egg consumption on the metabolic syndrome, which is characterized by dyslipidemia, hypertension, insulin resistance and large waist circumference. They applied a 4-week intervention of either three eggs per day or choline bitrartate supplements that induced similar plasma choline concentration. They found higher benefits of egg intake compared to choline supplementation in various biomarkers of participants with metabolic syndrome, such as reduced inflammation, insulin, insulin resistance and C-reactive protein. This was attributed to additional antioxidants in eggs. In spite of being a major source of dietary choline, egg consumption has been controversial because of their high cholesterol level. Interestingly, this study found no changes in total plasma cholesterol, triglycerides and fasting blood glucose, thus supporting eggs as a healthy food choice for people suffering with metabolic syndrome.

Likewise, prenatal choline influences the effects of obesity on metabolic dysregulation, as has been demonstrated in rodent models. Korsmo and coworkers [2], however, reported sexual dimorphism regarding the effect of choline supplementation in preventing the consequences of pre- and post-weaning obesogenic diets. While maternal choline supplementation had no effect on the metabolic outcomes of female offspring, the male offspring exhibited lower fasting blood glucose, better glucose tolerance, reduced leptin secretion and insulin upregulation. The authors discuss the females’ greater resistance to the metabolic disturbance induced by maternal obesity in terms of sex differences in hormonal, metabolic and gut microbiome regulation. Their results support the fact that early dietary choline can mitigate the deleterious postnatal consequences of maternal obesity, probably as a result of improved adipose tissue metabolism.

Finally, this Special Issue includes the first siblings metabolomic study in pediatric celiac disease. Celiac disease is an autoimmune enteropathy triggered by dietary gluten and related prolamins in genetically predisposed individuals. It is characterized by different degrees of intestinal inflammation. The metabolomics of this disease originate in alterations of the gut microbioma, intestinal permeability and absorption. A variety of changes in energy metabolism, lipid metabolism and microbiome-derived metabolites has been described. Among them, those changes that affect methionine, choline and choline-derived lipids point to the alteration of one-carbon metabolism. Martín-Masot and colleagues [4] compared children with celiac disease on a gluten-free diet with healthy control siblings. They did not find any difference in metabolites involved in choline metabolism and other pathways involved in one-carbon metabolism, except the transsulfuration pathway. In fact, a reduction in cysteine and cystathionine plasma content was observed. This suggests that the gluten-free diet is not able to eliminate the alterations in the transsulfuration pathway, in spite of reversing other metabolic alterations as well as clinical symptoms. Hence, this persistent metabolic alteration deserves further research in order to be proposed as a potential biomarker of this pathology.

The papers included in this Special Issue highlight the widespread involvement of choline in biological functions at different developmental stages as well as its relationship with pathological clinical conditions. The findings reported update our knowledge in the field and provide new insights based on animal and human research. Although further research is needed on choline involvement in health and disease, it is evident that advancing knowledge in this field will contribute to improving the general population’s eating patterns. Moreover, assessing the choline status in patients might help to develop biomarkers useful for diagnosis and the design of adequate dietary interventions. Taken together, these findings outline an interesting and promising scenario with regard to enhancing health care.

## References

[1] DiBella M., Thomas M.S., Alyousef H., Millar C., Blesso C., Malysheva O., Caudill M.A., Fernandez M.L. (2020). Choline Intake as Supplement or as a Component of Eggs Increases Plasma Choline and Reduces Interleukin-6 without Modifying Plasma Cholesterol in Participants with Metabolic Syndrome. Nutrients.

[2] Korsmo H.W., Edwards K., Dave B., Jack-Roberts C., Yu H., Saxena A., Salvador M., Dembitzer M., Phagoora J., Jiang X. (2020). Prenatal Choline Supplementation during High-Fat Feeding Improves Long-Term Blood Glucose Control in Male Mouse Offspring. Nutrients.

[3] Arias N., Arboleya S., Allison J., Kaliszewska A., Higarza S.G., Gueimonde M., Arias J.L. (2020). The Relationship between Choline Bioavailability from Diet, Intestinal Microbiota Composition, and Its Modulation of Human Diseases. Nutrients.

[4] Martín-Masot R., Mota-Martorell N., Jové M., Maldonado J., Pamplona R., Nestares T. (2020). Alterations in One-Carbon Metabolism in Celiac Disease. Nutrients.

[5] Gámiz F., Gallo M. (2021). A Systematic Review of the Dietary Choline Impact on Cognition from a Psychobiological Approach: Insights from Animal Studies. Nutrients.

[6] Moretti A., Paoletta M., Liguori S., Bertone M., Toro G., Iolascon G. (2020). Choline: An Essential Nutrient for Skeletal Muscle. Nutrients.

